# Quantitative image analysis using chest computed tomography in the evaluation of lymph node involvement in pulmonary sarcoidosis and tuberculosis

**DOI:** 10.1371/journal.pone.0207959

**Published:** 2018-11-26

**Authors:** Chang Un Lee, Semin Chong, Hye Won Choi, Jae Chol Choi

**Affiliations:** 1 Department of Radiology, Chung-Ang University Hospital, Chung-Ang University College of Medicine, Seoul, Korea; 2 Department of Radiology, Samsung Medical Center, Sungkyunkwan University School of Medicine, Seoul, Korea; 3 Division of Pulmonary Medicine, Department of Internal Medicine, Chung-Ang University Hospital, Chung-Ang University College of Medicine, Seoul, Korea; A.C. Camargo Cancer Center, BRAZIL

## Abstract

**Purpose:**

To evaluate the feasibility of quantitative analysis of chest computed tomography (CT) scans for the assessment of lymph node (LN) involvement in patients with pulmonary tuberculosis and sarcoidosis.

**Methods:**

In 47 patients with tuberculosis (n = 26) or sarcoidosis (n = 21), 115 lymph nodes (tuberculous, 55; sarcoid, 60) were visually analyzed on chest CT scans according to their size, location, attenuation and shape. Each node was manually segmented using image analysis tool, which was quantitatively analyzed using the following variables: Feret’s diameter, perimeter, area, circularity, mean grey value (Mean), standard deviation (SD) of grey value, minimum grey value (Min), maximum grey value (Max), median grey value (Median), skewness, kurtosis, and net enhancement. We statistically analyzed the visual and quantitative CT features of tuberculous and sarcoid LNs.

**Results:**

In visual CT analysis, the mean node size in sarcoidosis was significantly greater than that in tuberculosis. There were no statistical differences between tuberculous and sarcoid LNs in terms of location and shape. Central low attenuation and peripheral rim enhancement were more frequently observed in tuberculous LNs than in the sarcoid ones. In quantitative CT analysis, there were significant differences in the values of the Feret’s diameter, perimeter, area, circularity, mean grey value, SD, median, skewness, and kurtosis between tuberculous and sarcoid LNs.

**Conclusions:**

Quantitative CT analysis using CT parameters with pixel-by-pixel measurements can help to differentiate of tuberculous and sarcoid LNs.

## Introduction

Granulomatous lymphadenitis can be classified into infectious and non-infectious types. A typical example of infectious granulomatous lymphadenitis is tuberculous lymphadenitis and that of non-infectious lymphadenitis is sarcoidosis [1]. Tuberculous lymphadenitis, which presents with caseating granuloma as one of its pathognomonic manifestations, mainly affects either the cervical or mesenteric lymph nodes; however, the tuberculous infection has often been reported as invading the mediastinal and axillary lymph nodes as well [2]. In contrast, sarcoidosis is a systemic disease that most commonly affects intrathoracic lymph nodes. It presents non-caseating granuloma as a characteristic feature, and its exact etiology is unknown.

In particular, tuberculosis affects the right paratracheal or hilar lymph nodes, most frequently in the thoracic region. Tuberculous lymphadenitis is characterized as presenting low attenuation as well as peripheral rim enhancement on computed tomography (CT) images. Pathologically, central low attenuation indicates caseous necrosis, while peripheral rim enhancement indicates granulation tissue accompanying inflammation and hypervascularity [3]. In patients with sarcoidosis, the CT findings of lymph node involvement present as bilateral, symmetric enlargement of the hilar and right paratracheal lymph nodes and relatively uniform contrast enhancement. These two diseases can be distinguished pathologically on the basis of the presence of central caseating necrosis. However, radiographic differentiation of the two diseases is not often clear and easy because central caseating necrosis is not always visualized on CT images.

Most studies have investigated the involvement of mediastinal or hilar lymph nodes in tuberculosis or sarcoidosis by visual analysis of CT images. However, the efficacy of visual analysis in distinguishing tuberculous lymphadenitis and sarcoidosis might be limited in cases of tuberculous lymphadenitis without central low attenuation and peripheral rim enhancement.

In order to overcome the limitations of conventional visual analyses, a quantitative CT analysis method has been introduced, which allows the quantification and analysis of the differences in grey-scale images that might not be recognized by human eyes, using computer software. Many previous studies have applied quantitative CT analysis for the evaluation of lymph node involvement in various diseases [4, 5]. Bayanati et al. quantitatively evaluated mediastinal lymph node involvement in lung cancers by quantitative CT analysis using the grey-level co-occurrence and run-length matrix textural features [5]. They were able to diagnose lymph node involvement with a sensitivity of 81% and specificity of 80%; they also reported accuracies of 84% and 71% for the diagnosis of malignant and benign lymph nodes, respectively. To the best of our knowledge, however, there have been no studies till date of quantitative CT analysis using computer software for the differential diagnosis of tuberculous lymphadenitis and sarcoidosis. Therefore, in the present study, we have evaluated the feasibility of quantitative CT analysis of chest CT scans using computer software for the assessment of lymph node involvement in patients with pulmonary tuberculosis and sarcoidosis.

## Materials and methods

The institutional review board of our institution approved this retrospective study (C2016062 (1805)) and waived the requirement for informed patient consent for inclusion in this study.

A search of our institutional electronic medical records database yielded the data of 580 patients with either thoracic tuberculosis (n = 521) or sarcoidosis (n = 59). Patients with tuberculosis had been diagnosed between January 2009 and January 2014 by polymerase chain reaction analysis, bacterial culture, or biopsy, while those with sarcoidosis had been diagnosed between April 2006 and January 2015 by pathological analysis by thoracic lymph node or lung biopsy. Of the 580 patients, 414 with either thoracic tuberculosis (n = 393) or sarcoidosis (n = 21) who had undergone contrast-enhanced chest CT (Brilliance 64 and Brilliance iCT 256, Philips Healthcare, Cleveland, OH, USA; LightSpeed Pro 16 and Optima 660, GE Medical Systems, Milwaukee, WI, USA) were initially selected. Of the 414 patients, those presenting either mediastinal lymph nodes with short-axis diameter > 1 cm or hilar lymph nodes with short or long-axis diameters > 1 cm were selected. Thus, finally, 47 patients with tuberculosis (n = 26) or sarcoidosis (n = 21) were included in this study.

A total of 115 lymph nodes (tuberculous, 55; sarcoid, 60) in the CT images of the 47 patients were visually analyzed in terms of their size, location, attenuation, and shape. The sizes of the hilar and mediastinal lymph nodes were evaluated by measurement of the longest diameter along any of the axes and the short axis, respectively, using the Picture Archiving and Communication System (PACS; Maroview, Infinitt technology, Seoul, Korea). The locations of the lymph nodes were classified into 14 regions, and the lymph nodes were then categorized based on location into four groups—the superior mediastinal (right or left paratracheal, pre-vascular, and retrotracheal), inferior mediastinal (subcarinal, paraesophageal, and pulmonary ligament), aortic (subaortic and para-aortic), and hilar lymph nodes—according to the lymph node map of the International Association for the Study of Lung Cancer (IASLC) [6]. The attenuation of the lymph nodes was assessed in terms of the presence or absence of central low attenuation with peripheral rim enhancement. In terms of shape, lymph nodes with short-to-long diameter ratios > 1.5:1 were classified as ovoid lymph nodes, and the rest were classified as round nodes.

**Fig 1:**
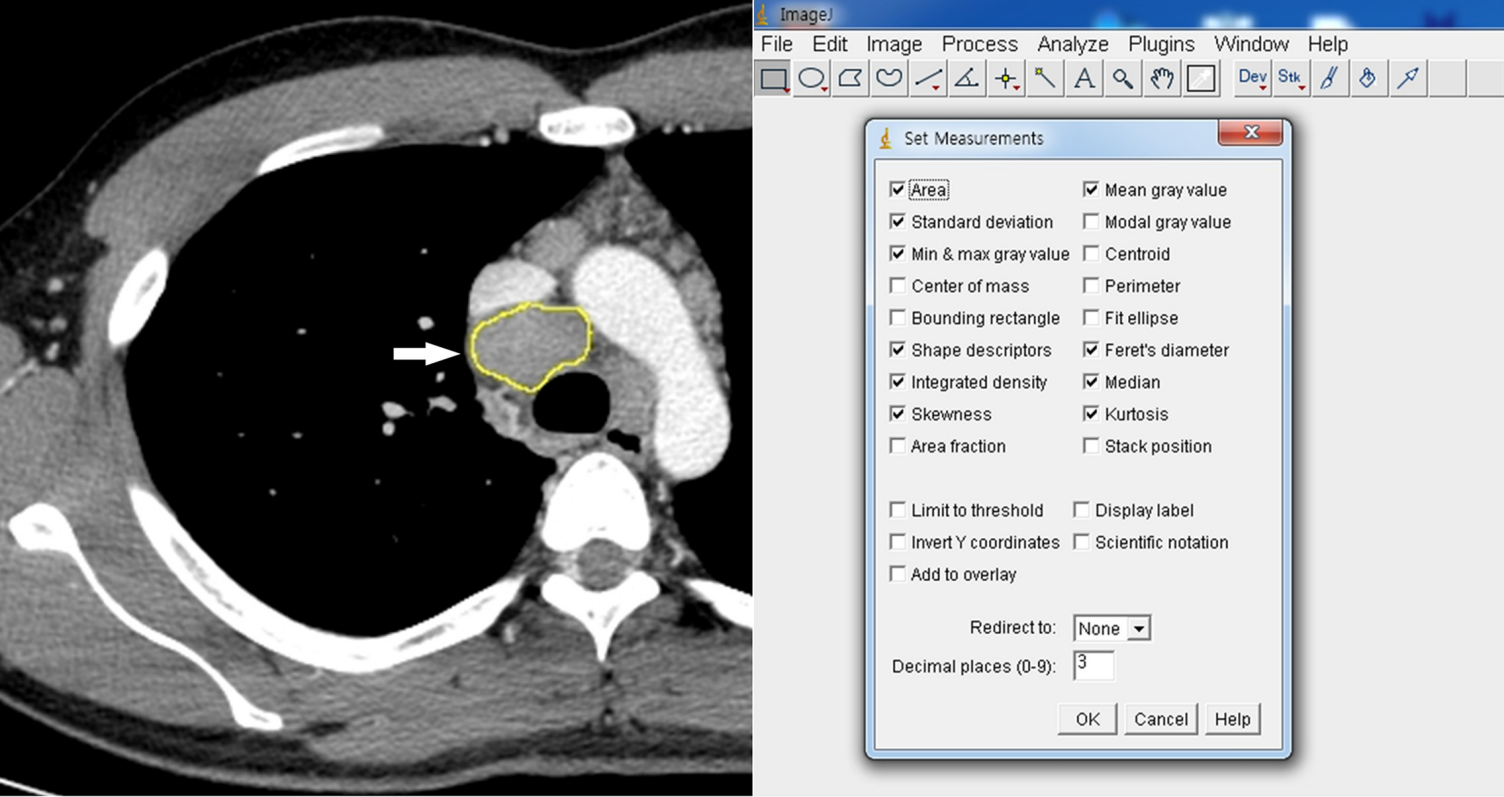
2D CT segmentation of the region of interest and quantitative CT analysis. (A) The lymph node of interest (arrow) was manually segmented on the image processing and analysis software using a graphic touch pen. (B) The evaluated measurements were specified in the dialog box of the image processing and analysis software.

The CT images of the 115 lymph nodes were converted into digital imaging and communication in medicine (DICOM) files and transferred to image analysis tool (ImageJ, National Institutes of Health, Bethesda, MD, USA) [7].The target node was manually segmented using a graphic touch pen in order to extract the region of interest (ROI) (Fig 1). The ROIs thus extracted were quantitatively analyzed using the following variables [8]:

Feret’s diameter (Feret’s): The longest distance between any two points along the selection boundary; also known as the maximum caliper
Perimeter: Length of the outside boundary of the selection
Area: Area of the selection in square pixels
Circularity: $4\;\pi \times \frac{area}{{perimeter}^{2}}$, with a value of 1.0 indicating a perfect circle
Mean grey value (Mean): Average grey value within the selection
Standard deviation (SD): Standard deviation of the grey values used to calculate the mean grey value
Minimum grey value (Min): Minimum grey value within the selection
Maximum grey value (Max): Maximum grey value within the selection
Median: Median value of the pixels in the image or selection
Skewness: Third order moment about the mean
Kurtosis: Fourth order moment about the mean
Net enhancement value: Mean grey value of a contrast-enhanced image minus the mean grey value of a non-contrast enhanced image

All of the tuberculous and sarcoid lymph nodes were sub-classified according to presence or absence of lung involvement on the basis of both visual and quantitative CT image analyses by blinded method without clinical information.

We statistically analyzed the visual and quantitative CT features of the tuberculous and sarcoid lymph nodes using the Mann-Whitney test and the Student’s t-test, chi-square test, or Fisher’s exact test. Statistical analysis was performed using PASW Statistics for Windows, Version 18.0 (SPSS Inc., Chicago, IL, USA). A p value < 0.05 was considered statistically significant.

## Results

Table 1 shows the results of visual CT image analysis of lymph nodes. The average size of the lymph nodes in patients with sarcoidosis was 16.6 mm, which was statistically significantly greater than that in patients with tuberculosis (13.3 mm; p < 0.001). There were no statistical differences between the tuberculous and sarcoid lymph nodes in terms of location (p = 0.091), and most lymph nodes were present in the superior mediastinal and hilar node groups. Central low attenuation and peripheral rim enhancement were more frequently observed in tuberculous lymph nodes than in the sarcoid ones (p < 0.001) and in cases with tuberculosis with lung involvement than in those without lung involvement (p = 0.036).There were no significant differences in the shapes of lymph nodes between tuberculosis and sarcoidosis (p = 0.230). However, sarcoidosis with lung involvement showed a significantly higher frequency of round-shaped lymph nodes than that without lung involvement (p = 0.009); there were no significant differences in the shapes of lymph nodes between tuberculosis with or without lung involvement.

**Table 1:** Visual CT Analysis of lymph nodes between tuberculosis and sarcoidosis with or without lung involvement.

	Total (n = 115)	TB (n = 55)	SA (n = 60)	P value	TB0 (n = 17)	TB1 (n = 38)	P value	SA0 (n = 30)	SA1 (n = 30)	P value
Mean size (mm)	15	13.3	16.6	<0.001	12.4	13.7	0.211	15.7	17.5	0.083
Location (group)				0.091			0.876			0.752
Superior mediastinal	50	30 (55)	20 (33)		9 (53)	21 (55)		12 (40)	8 (27)	
Inferior mediastinal	20	9 (16)	11 (18)		2 (12)	7 (18)		5 (17)	6 (20)	
Aortic	15	4 (7)	11 (18)		1 (6)	3 (8)		5 (17)	6 (20)	
Hilar	30	12 (22)	18 (30)		5 (29)	7 (18)		8 (27)	10 (33)	
Central low attenuation				<0.001			0.024			0.237
Presence	36	33 (60)	3 (5)		14 (82)	19 (50)		3 (10)	0 (0)	
Absence	79	22 (40)	57 (95)		3 (18)	19 (50)		27 (90)	30 (100)	
Shape				0.230			0.670			0.009
Round	56	30 (55)	26 (43)		10 (59)	20 (53)		8 (27)	18 (60)	
Oval	59	25 (45)	34 (57)		7 (41)	18 (47)		22 (73)	12 (40)	

TB: Tuberculosis SA: Sarcoidosis TB0: Tuberculosis without lung involvement TB1: Tuberculosis with lung involvement SA0: Sarcoidosis without lung involvement SA1: Sarcoidosis with lung involvement NA = not available () = %

**Fig 2:**
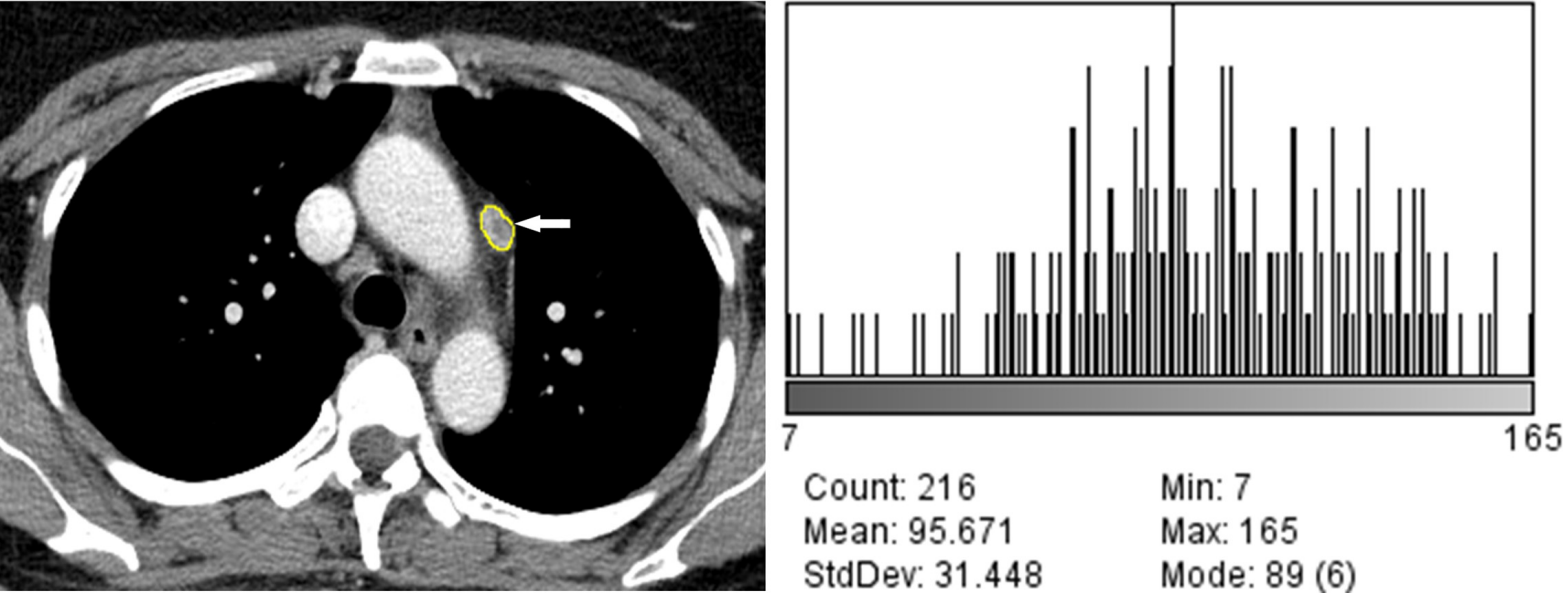
49-year-old woman with tuberculous lymphadenopathy without lung involvement. (A) Mediastinal window image of contrast-enhanced chest CT scan shows a segmented subaortic lymph node of the aortic group (arrow). (B) Histogram plot shows the CT data profile of the segmented lymph node (skewness, -0.43; kurtosis, 0.56).

**Fig 3:**
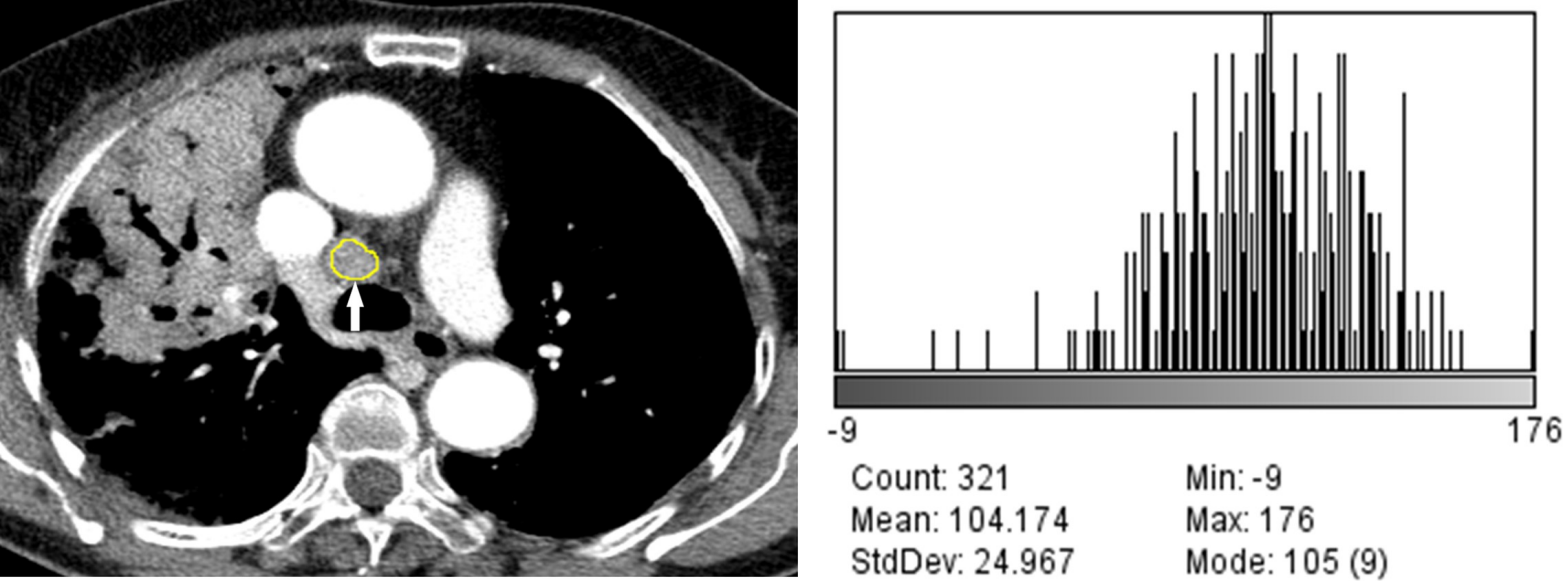
76-year-old woman with tuberculous lymphadenopathy with lung involvement. (A) Mediastinal window image of contrast-enhanced chest CT scan shows a segmented right lower paratracheal lymph node (arrow). (B) Histogram plot shows the CT data profile of the segmented lymph node (skewness, -1.14; kurtosis, 2.86).

**Fig 4:**
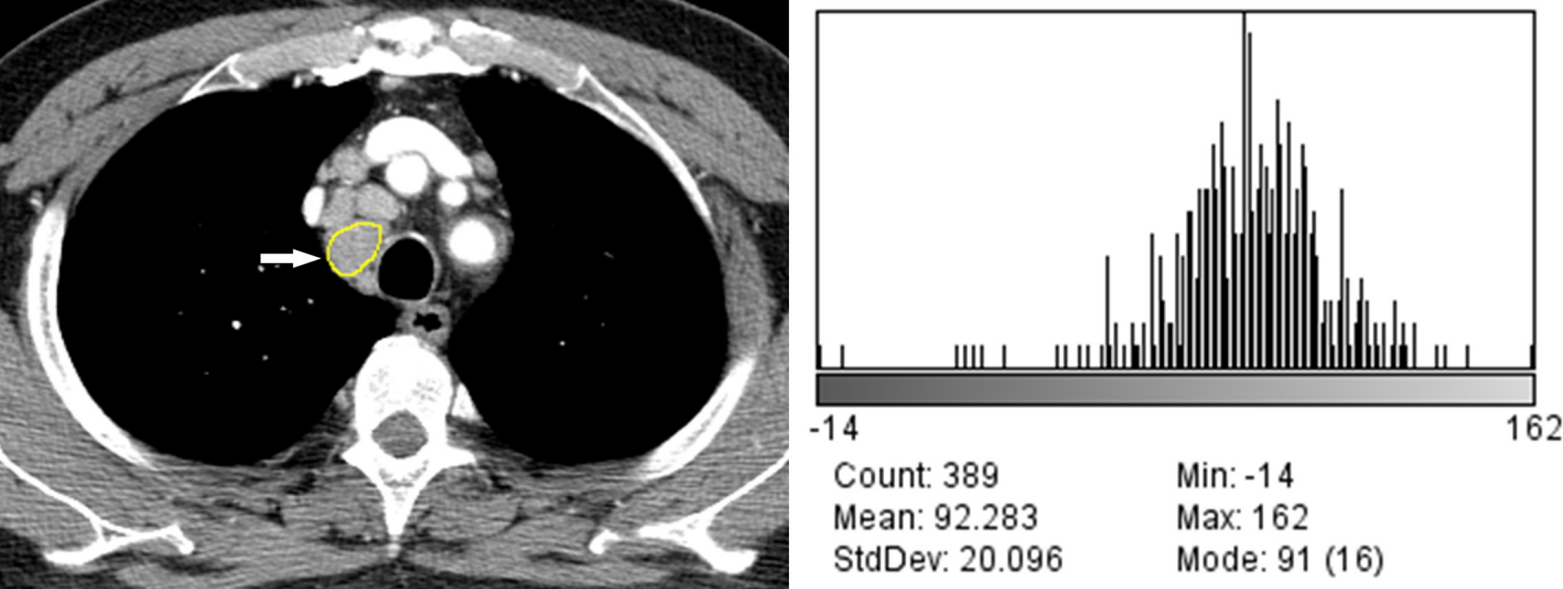
70-year-old man with sarcoidosis without lung involvement. (A) Mediastinal window image of contrast-enhanced chest CT scan shows a segmented right upper paratracheal lymph node (arrow). (B) Histogram plot shows the CT data profile of the segmented lymph node (skewness, -1.16; kurtosis, 5.75).

**Fig 5:**
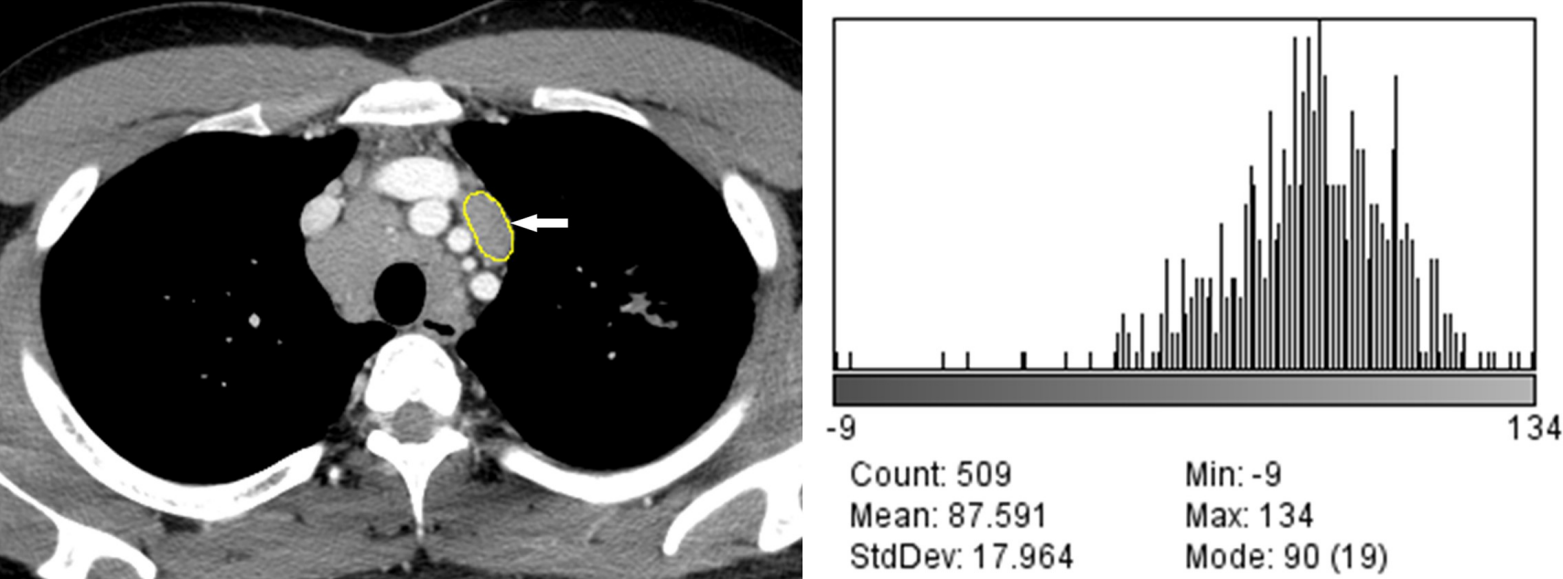
33-year-old man with sarcoidosis with lung involvement. (A) Mediastinal window image of contrast-enhanced chest CT scan shows a segmented prevascular lymph node (arrow). (B) Histogram plot shows the CT data profile of the segmented lymph node (skewness, -1.56; kurtosis, 5.26).

The results of quantitative CT image analysis of the tuberculous and sarcoid lymph nodes are presented in Table 2. There were significant differences in the values of the Feret’s diameter, perimeter, area, circularity, mean grey value, SD, median, skewness, and kurtosis between the tuberculous and sarcoid lymph nodes (p < 0.05). There were significant differences in the Feret’s diameter, area, mean grey value, SD, minimum and maximum grey values, kurtosis, and net enhancement of the lymph nodes between patients with tuberculosis with and without lung involvement (Figs 2 and 3). In patients with sarcoidosis with and without lung involvement, the lymph nodes showed significant differences between the two groups in terms of the perimeter, area, mean grey value, SD, median grey value, skewness, and net enhancement (Figs 4 and 5).

**Table 2:** Quantitative CT analysis of mediastinal and hilar lymph nodes in tuberculosis and sarcoidosis with or without lung involvement.

	Total	Tuberculosis	Sarcoidosis	P value	TB		P value	SA		P value
					TB0	TB1		SA0	SA1	
Feret’s diameter (mm)	19.00	16.66	21.15	<0.001	14.96	17.42	0.045	20.02	22.28	0.064
Perimeter (mm)	55.63	47.30	63.27	<0.001	42.71	49.35	0.056	58.52	68.01	0.006
Area (mm^2^)	199.15	153.12	241.34	<0.001	129.42	163.73	0.041	208.32	274.36	0.012
Circularity	0.77	0.82	0.73	<0.001	0.82	0.81	0.651	0.74	0.72	0.300
Mean (HU)	72.14	69.71	74.37	0.040	58.67	74.64	0.041	81.37	67.37	0.006
SD (HU)	29.00	36.27	22.34	0.005	20.80	43.19	<0.001	24.89	19.79	0.007
Min (HU)	-34.50	-21.38	-46.52	0.409	4.65	-33.03	0.001	-64.17	-28.87	0.145
Max (HU)	161.92	190.49	135.73	0.628	114.82	224.42	0.006	142.43	129.03	0.055
Median (HU)	70.45	64.53	75.88	0.003	57.88	67.50	0.087	83.67	68.10	0.003
Skewness	-0.44	-0.02	-0.83	<0.001	0.13	-0.08	0.113	-1.22	-0.43	0.003
Kurtosis	3.60	1.60	5.42	<0.001	-0.19	2.41	0.007	7.61	3.23	0.092
Net enhancement(HU)	32.57	31.71	33.35	0.495	12.54	40.29	0.007	39.15	27.55	0.019

TB0: Tuberculosis without lung involvement TB1: Tuberculosis with lung involvement SA0: Sarcoidosis without lung involvement SA1: Sarcoidosis with lung involvement Feret’s: Feret’s diameter Mean: Mean grey value SD: Standard deviation Min: Minimum grey value Max: Maximum grey value Median: Median grey value

The results of quantitative CT image analysis of lymph nodes without central low attenuation are summarized in Table 3. There were statistically significant differences in the Feret’s diameter, perimeter, area, and circularity between the tuberculous and sarcoid lymph nodes without central low attenuation, regardless of lung involvement. There were no statistically significant differences in any of the quantitative CT parameters between tuberculous lymph nodes with and without lung involvement. However, sarcoid lymph nodes and without lung involvement exhibited statistically significant differences in terms of perimeter, area, mean grey value, SD, median, and skewness. In cases of tuberculosis and sarcoidosis without lung involvement, there were statistically significant differences in the Feret’s diameter, skewness, and kurtosis between the two diseases. In cases of tuberculosis and sarcoidosis with lung involvement, there were statistically significant differences in the Feret’s diameter, perimeter, area, circularity, mean grey value, SD, and net enhancement between the two diseases.

**Table 3:** Quantitative CT Analysis of mediastinal and hilar lymph nodes without central low attenuation in tuberculosis and sarcoidosis with or without lung involvement.

	TB	SA	P value	TB0	TB1	P value	SA0	SA1	P value	P value	
										TB0 vs SA0	TB1 vs SA1
Feret’s diameter (mm)	18.32	21.23	0.004	16.37	18.63	0.226	20.08	22.28	0.086	<0.001	0.003
Perimeter (mm)	51.98	63.54	<0.001	48.64	52.51	0.523	58.58	68.00	0.009	0.221	<0.001
Area (mm^2^)	172.11	243.30	<0.001	150.81	175.47	0.495	208.79	274.36	0.019	0.285	<0.001
Circularity	0.79	0.73	<0.001	0.80	0.79	0.770	0.74	0.72	0.347	0.109	0.002
Mean (HU)	87.18	73.00	0.146	70.84	89.76	0.356	79.25	67.37	0.019	0.392	0.027
SD (HU)	51.69	22.28	0.066	21.25	56.49	0.586	25.04	19.79	0.010	0.468	0.004
Min (HU)	-25.64	-47.82	0.638	6.00	-30.63	0.260	-68.89	-28.87	0.139	0.117	0.545
Max (HU)	287.0	134.28	0.262	131.33	311.58	0.523	140.11	129.03	0.121	0.559	0.071
Median (HU)	75.82	74.53	0.787	71.33	76.53	0.594	81.67	68.10	0.010	0.314	0.140
Skewness	-0.16	-0.83	0.207	-0.05	-0.18	0.897	-1.28	-0.43	0.004	0.020	0.878
Kurtosis	3.87	5.16	0.983	-0.05	4.49	0.053	7.74	2.83	0.079	0.026	0.151
Net enhancement (HU)	44.22	31.96	0.146	21.59	47.79	0.258	36.85	27.55	0.063	0.151	0.020

TB0: Tuberculosis without lung involvement TB1: Tuberculosis with lung involvement SA0: Sarcoidosis without lung involvement SA1: Sarcoidosis with lung involvement Feret’s: Feret’s diameter Mean: Mean grey value SD: Standard deviation Min: Minimum grey value Max: Maximum grey value Median: Median grey value

The results of quantitative CT image analysis of lymph nodes with central low attenuation are summarized in Table 4. There were statistically significant differences in circularity, mean and maximum grey values, median and skewness of lymph nodes between tuberculosis and sarcoidosis, regardless of lung involvement. There were statistically significant differences in the SD, minimum and maximum grey values, and net enhancement between tuberculous lymph nodes with and without lung involvement. In cases of tuberculosis and sarcoidosis with no lung involvement, there were statistically significant differences in circularity, mean and maximum grey values, median, skewness and net enhancement. None of the lymph nodes in cases of sarcoidosis with lung involvement exhibited central low attenuation.

**Table 4:** Quantitative CT Analysis of lymph nodes with central low attenuation in tuberculosis and sarcoidosis with or without lung involvement.

	TB	SA	P value	TB0	TB1	SA0	P value	
							TB0 vs TB1	TB0 vs SA0
Feret’s diameter (mm)	15.56	19.46	0.139	14.66	16.22	19.46	0.314	0.099
Perimeter (mm)	44.18	58.00	0.070	41.44	46.20	58.00	0.288	0.056
Area (mm^2^)	140.46	204.05	0.206	124.84	151.98	204.05	0.361	0.172
Circularity	0.83	0.75	0.005	0.83	0.84	0.75	0.441	0.046
Mean (HU)	58.06	100.43	0.009	56.06	59.52	100.43	0.566	0.001
SD (HU)	25.99	23.49	0.074	20.70	29.89	23.49	<0.001	0.360
Min (HU)	-18.55	-21.67	0.787	4.36	-35.42	-21.67	<0.001	0.408
Max (HU)	126.15	163.33	0.037	111.29	137.10	163.33	0.009	0.012
Median (HU)	57.00	101.67	<0.001	55.00	58.47	101.67	0.595	0.001
Skewness	0.08	-0.72	0.008	0.17	0.01	-0.72	0.317	0.003
Kurtosis	0.09	2.95	0.066	-0.23	0.32	2.95	0.529	0.339
Net enhancement(HU)	23.37	59.83	0.058	10.60	32.78	59.83	0.046	0.036

TB0: Tuberculosis without lung involvement TB1: Tuberculosis with lung involvement SA0: Sarcoidosis without lung involvement SA1: Sarcoidosis with lung involvement Feret’s: Feret’s diameter Mean: Mean grey value SD: Standard deviation Min: Minimum grey value Max: Maximum grey value Median: Median grey value

## Discussion

To date, most studies have evaluated lymph nodes by means of visual assessment of their size and shape on CT scans. For instance, loss of normal oval shape, increase in short-axis diameter, unclear boundaries, and presence of central low attenuation were often used as indicators for the diagnosis as well as differentiation of lymph node diseases. However, visual CT image analysis on the basis of shape and size of lymph nodes exhibits significant limitations in the diagnosis of lymph node metastasis as well as infectious and inflammatory lymph node diseases. In order to overcome these limitations, FDG PET/CT, a technique that can assess cellular metabolic activity, was introduced. However, the superiority of this technique over visual CT image analysis in terms of specificity is debated. In addition, several studies evaluated the efficacies of quantitative analyses of CT and ultrasonography (US) images in the differentiation of metastatic and benign lymph node diseases [5, 9, 10]. In the present study, we quantitatively analyzed the CT images of mediastinal and hilar lymph nodes in patients with tuberculosis and sarcoidosis using image analysis software. To the best of our knowledge, this is the first study on quantitative CT image analysis of lymph nodes in patients with tuberculosis and sarcoidosis.

In a comparative study of the average size of lymph nodes on CT images between tuberculosis and sarcoidosis, Dhooria et al. reported no significant differences in the short-axis diameters of lymph nodes between the two diseases [11]. In contrast, in the present study, the results of visual CT image analysis revealed that the average size of lymph nodes in sarcoidosis was statistically significantly greater compared to that in tuberculosis; further, the values of the Feret’s diameter, perimeter and area of the lymph nodes were statistically significantly greater in sarcoidosis than in tuberculosis, according to the results of quantitative CT image analysis. This indicates that there are differences in the one and two-dimensional measurements of lymph node size between both diseases. Therefore, it might be possible to perform a more objective and accurate assessment of the two diseases by evaluating the various CT quantification parameters associated with size.

Computer-based quantitative analysis of CT images, which can be used to assess minimal changes in pixel units that might not be recognized by human eyes, has quickly emerged as a feasible diagnostic tool. In the present study, there were statistically significant differences in the skewness and kurtosis of the CT profile histograms between the tuberculosis and sarcoidosis groups (Table 2).The CT profile histogram of the sarcoidosis group showed statistically significantly lower negative skewness and higher positive kurtosis than that of the tuberculosis group. The skewness represents specific values of the asymmetric direction of a histogram in comparison to the average value as well as the degree of asymmetry. Positive skewness indicates a right-sided histogram, while negative values indicate a left-sided histogram. In contrast, kurtosis indicates the sharpness of a histogram. Generally speaking, values between 0 and 3 indicate even (or flat) distribution, with 3 indicating normal height. Values of kurtosis > 3 indicate sharp distribution of values [12]. In the present study, the CT values of the sarcoidosis group exhibited sharper distribution compared to those of the tuberculosis group. In addition, the CT profile histograms of both groups exhibited negative skewness, indicating left-sided data distribution in both groups (Figs 2–5). We believe that the skewness and kurtosis values of the histograms of CT images may be useful as CT quantification parameters in the differentiation of tuberculosis and sarcoidosis in terms of evaluation of lymph node involvement.

In cases with no central low attenuation, it is difficult to differentiate between tuberculous and sarcoid lymph nodes by visual CT image analysis. However, in the present study, the results of quantitative CT image analysis of lymph nodes with no central low attenuation revealed statistically significant differences in the Feret’s diameter, perimeter, area, and circularity of lymph nodes between tuberculosis and sarcoidosis. Therefore, despite their limitations in the accurate evaluation of pathological and clinical significances, we believe that these quantitative variables could be potentially used for the differentiation of tuberculosis and sarcoidosis.

There are several limitations of our study. First, there is a possibility of selection bias because of the retrospective nature of our study as well as the small sample size. Consequently, imaging data, such as the scanning parameters for chest CT image acquisition (e.g., radiation dose, slice thickness, and contrast-enhancement time), were not homogenous. Second, not all of the lymph nodes were pathologically evaluated in order to confirm the diagnosis of tuberculosis or sarcoidosis. Although pathological lymph nodes were selected for inclusion in our study on the basis of their size, the possibility of lymph node enlargement unrelated to either tuberculosis or sarcoidosis was not considered. Third, the lymph nodes were manually segmented using a touch pen. Although the accuracy of segmentation was finally confirmed by a thoracic radiologist with 10 years of experience in thoracic imaging, there is a possibility of measurement error. In the future, semi or fully automated segmentation using image analysis software with much improved algorithms might prove feasible for quantitative CT image analysis of lymph nodes. Finally, we did not include the performance test of the quantitative CT image analysis, with sensitivity, specificity and accuracy in the differentiation between the two granulomatous diseases. However, this quantitative CT analysis is likely to be useful in the evaluation of visually normal lymph nodes and is expected in future studies.

In conclusion, we investigated the feasibility of various CT quantification parameters for the differentiation of tuberculous and sarcoid lymph nodes. To our knowledge, the present study was the first to perform quantitative analysis of imaging data extracted from the CT images of mediastinal and hilar lymph nodes in pulmonary tuberculosis and sarcoidosis, using image analysis software. We believe that quantitative CT image analysis for the measurement of fine pixel units, which might not be recognized by human eyes, can help identify CT quantification parameters for the differentiation of tuberculous and sarcoid lymph nodes.
